# Intra-colony channels in *E. coli* function as a nutrient uptake system

**DOI:** 10.1038/s41396-020-0700-9

**Published:** 2020-06-17

**Authors:** Liam M. Rooney, William B. Amos, Paul A. Hoskisson, Gail McConnell

**Affiliations:** 1 Strathclyde Institute of Pharmacy and Biomedical Sciences, University of Strathclyde, 161 Cathedral Street, Glasgow, G4 0RE UK; 2 Department of Physics, SUPA, University of Strathclyde, 107 Rottenrow East, Glasgow, G4 0NG UK; 3 Present Address: Institute of Biological Chemistry, Biophysics and Bioengineering, School of Engineering and Physical Sciences, Heriot-Watt University, Edinburgh, EH14 4AS UK

**Keywords:** Biofilms, Microbial ecology

## Abstract

The ability of microorganisms to grow as aggregated assemblages has been known for many years, however their structure has remained largely unexplored across multiple spatial scales. The development of the Mesolens, an optical system which uniquely allows simultaneous imaging of individual bacteria over a 36 mm^2^ field of view, has enabled the study of mature *Escherichia coli* macro-colony biofilm architecture like never before. The Mesolens enabled the discovery of intra-colony channels on the order of 10 μm in diameter, that are integral to *E. coli* macro-colony biofilms and form as an emergent property of biofilm growth. These channels have a characteristic structure and re-form after total mechanical disaggregation of the colony. We demonstrate that the channels are able to transport particles and play a role in the acquisition of and distribution of nutrients through the biofilm. These channels potentially offer a new route for the delivery of dispersal agents for antimicrobial drugs to biofilms, ultimately lowering their impact on public health and industry.

## Introduction

Bacteria frequently grow as surface-attached communities within complex extracellular matrices containing extracellular polysaccharides (EPS), lipids, proteins and nucleic acids [1, 2]. These microbial communities may be composed of one or more species (mono/poly-microbial) and are found in almost every ecological niche [3]. The structure and protective matrix enveloping the biofilm confers resistance to unfavourable environmental conditions and deleterious agents such as biocides or antibiotics [4–8]. The ecological benefits of these heterogeneous phenotypic responses are poorly understood, yet there is evidence that suggests that biofilm formation may promote the development and spread of antimicrobial resistance [9]. Consequently, the study of biofilm structure is vital in understanding how microbial assemblages grow and persist in a range of environments and conditions. The 3D organisation of biofilms may take many forms [10–13]; for example, mushroom-shaped biofilms grown in liquid flow systems, thin sheet-like biofilms in static liquid systems, pellicle biofilms grown at liquid/air interfaces and macro-colony biofilms on solid surfaces. Although morphologically distinct, what classifies these structurally different communities as ‘biofilms’ lies with their shared fundamental biochemical signals and pathways [3].

Dynamic computational modelling programmes, such as CellModeller [14, 15], have been used to predict the spatial patterning and arrangement of cells within bacterial communities [14–18]. In silico models primarily show growth of poly-microbial communities where cell shape, size, surface properties and cell–cell interactions influence the spatial organisation of the mature biofilm, resulting in sectoring of different strains into distinct populations, which has been validated experimentally [19–23]. However, in silico modelling has shown little evidence of structural ordering or complex spatial patterning largely as a result of a lack of effective multi-scale imaging techniques.

The study of living biofilms has been mainly performed by optical imaging, and has shown phenomena such as the density-dependent phage sensitivity in *Escherichia coli* colonies [24], the effect of pH on biofilms present on human tooth enamel [25] and the synchronies of growth and electrical signalling between adjacent bacterial colonies [26]. Observation by optical microscopy has also been used to investigate macroscopic channel features in biofilms. For example, the macro-colony folds formed by *Pseudomonas spp*. in response to oxygen stress when the biofilm reaches a critical mass [27, 28], the crenulations formed by *Bacillus spp*. macro-colonies for water transport [29, 30], or the cavernous water channels which are formed at the base of submerged mushroom-shaped biofilms often grown in under shear flow [31]. These studies have exposed a gap in the repertoire of the optical microscope in that either microbes could be imaged individually with a high-power objective lens, or the overall biofilm structure could be viewed at low magnification with poor resolution, particularly in depth, that individual microbial cells could not be seen. To address this shortcoming, we use the Mesolens to image intact live macro-colony biofilms in situ with isotropic sub-cellular resolution. In essence, the Mesolens is a giant objective lens with the unique combination of ×4 magnification with a numerical aperture (NA) of 0.47; which is approximately five-times greater than that of a conventional 4× objective lens [32]. The low magnification coupled with a high NA result in a field of view (FOV) measuring ~6 mm^2^ with lateral resolution of 700 nm and 7 μm axially, while the lens prescription provides a working distance of 3 mm. Moreover, the lens is chromatically corrected across the visible spectrum and designed to be compatible with various immersion routines. While the Mesolens has proven to be a powerful tool in neuroscience, developmental biology and pathology [32–34], it remains an untapped technology for biofilm imaging, where we can image whole live microbial communities with unprecedented detail within a single dataset without additional processing or stitching and tiling.

We used the Mesolens to investigate the internal architecture of mature *E. coli* macro-colony biofilms. We identified and characterised a previously undocumented channel system within these biofilms that facilitates nutrient uptake from the external environment and offers novel insight into nutrient delivery in large microbial communities. These findings offer additional support for diffusion dynamics in bacterial biofilms; which is widely accepted as the main route of delivery for any external compounds to enter a biofilm, whether they be nutrients or antimicrobial drugs [14–17]. In addition, we demonstrated that intra-colony channels form as an emergent property of biofilm formation in *E. coli*. These findings provide novel understanding of how spatial organisation in bacterial biofilms contributes to their ability to transport material from the external environment.

## Materials and methods

### Designing and 3D-printing a chamber slide for biofilm imaging

A custom imaging chamber was designed using AutoCAD (Autodesk, USA) with the purpose of imaging large-scale-cultured bacterial communities in situ using the Mesolens. The design consisted of a plate with dimensions 90 × 80 × 12 mm and a central well measuring 60 mm in diameter with a depth of 10 mm (Supplementary Fig. 1). The imaging chamber was 3D-printed using black acrylonitrile butadiene styrene plastic (FlashForge, Hong Kong) with a FlashForge Dreamer 3D printer (FlashForge, Hong Kong). The chamber slide was sterilised prior to use with 70% ethanol and UV irradiation for 15 min.

### Bacterial strains and growth conditions

All experiments were performed using the *E. coli* strains outlined in Supplementary Table 1. Colony biofilms were grown by inoculating a lawn of cells at a density of 1 × 10^4^ cfu/ml on either solid LB medium or M9 minimal medium [35] to achieve single colonies, and growth medium was supplemented with the appropriate selective antibiotic to maintain the photoprotein. The colonies were grown in the 3D-printed imaging mould at 37 °C for 18–24 h in darkened conditions prior to imaging. All experiments were repeated in triplicate to ensure observations were reliable.

### Specimen preparation

For colony imaging alone, colonies were submerged in sterile LB broth (refractive index (*n*) = 1.338) as a mounting medium following the allocated growth time prior to imaging. A large coverglass was placed over the central well of the imaging mould (70 × 70 mm, Type 1.5, 0107999098 (Marienfeld, Lauda-Koenigshofen, Germany)), and the colonies were then imaged using either the Mesolens or a conventional widefield epi-fluorescence microscope to compare their performance and to justify using the Mesolens to study biofilm architecture over conventional techniques.

The refractive index of the LB mounting medium was measured using an Abbe Refractometer (Billingham & Stanley Ltd, UK) which was calibrated using Methanol at 21 °C.

### Conventional widefield epi-fluorescence microscopy

Colony biofilms were imaged on a conventional Eclipse E600 upright widefield epi-fluorescence microscope (Nikon, Japan) equipped with a 4×/0.13 NA Plan Fluor objective lens (Nikon, Japan). GFP excitation was provided by a 490 nm LED from a pE-2 illuminator (CoolLED, UK), and emission was detected using a bandpass filter (BA 515–555 nm, Nikon, Japan) placed before an ORCA-spark digital CMOS camera (Hamamatsu, Japan). The camera detector was controlled using WinFluor software [36]. Colonies were imaged after 20 h of growth in an imaging mould as described above.

### Widefield epi-fluorescence mesoscopy

Specifications of the Mesolens have been previously reported [32], and therefore only the imaging conditions used in this study will be outlined here. GFP excitation was achieved using a 490 nm LED from a pE-4000 LED illuminator (CoolLED, UK). A triple-bandpass filter which transmitted light at 470 ± 10, 540 ± 10 and 645 ± 50 nm was placed in the detection pathway. The emission signal was detected using a VNP-29MC CCD camera with a chip-shifting modality (Vieworks, South Korea) to capture the full FOV of the Mesolens at high resolution. Widefield mesoscopic imaging was carried out using water immersion (*n* = 1.33) with the Mesolens’ correction collars set accordingly to minimise spherical aberration through refractive index mismatch.

### Confocal laser-scanning mesoscopy

For laser-scanning confocal mesoscopy, specimens were prepared as outlined above. Fluorescence excitation of GFP was obtained using the 488 nm line set at 5 mW from a multi-line LightHUB-4 laser combiner (Omicron Laserage, Germany). The green emission signal was detected using a PMT (P30-01, Senstech, UK) with a 550 nm dichroic mirror (DMLP550R, Thorlabs, USA) placed in the emission path and a 525/39 nm bandpass filter (MF525-39, Thorlabs, USA) placed before the detector.

For reflection confocal mesoscopy, incident light was sourced from a 488 nm line set at 1 mW from a multi-line LightHUB-4 laser combiner (Omicron Laserage, Germany). Reflected signal was detected using a PMT (P30-01, Senstech, UK) with no source-blocking filter in place.

Confocal laser-scanning mesoscopy was carried out using type DF oil immersion (*n* = 1.51) with the Mesolens’ correction collars set accordingly to minimise spherical aberration through refractive index mismatch.

### Structural assessment of intra-colony channels

To characterise the structure of intra-colony channels we sought to visualise the distribution of several archetypal structural components of biofilms.

As the biofilms in this study were submerged during imaging in a medium with known refractive index, we were able to determine if channels were filled with substances of differing refractive index (e.g., air) using reflection confocal mesoscopy as above. Solid LB was cast into a 3D-printed imaging chamber and inoculated with JM105 at a density of 1 × 10^4^ cfu/ml and incubated for 18–24 h at 37 °C in darkened conditions. Biofilms were mounted in sterile LB medium (*n* = 1.338) prior to imaging.

We then imaged the distribution of non-viable cells in the biofilm based on the approach developed by Asally [30]. Briefly, JM105-miniTn7-*HcRed1* colony biofilms were grown for imaging in 3D-printed imaging moulds as outlined previously. LB medium was supplemented with gentamicin (20 μg/ml) and 0.5 μM Sytox green dead-cell stain (S7020, Invitrogen, USA). Cells were seeded at a density of 1 × 10^4^ cfu/ml and grown for 18–24 h prior to imaging on the Mesolens in widefield epi-fluorescence mode as described above. A 490 and 580 nm LED from a pE-4000 LED illuminator (CoolLED, UK) were used to excite Sytox Green and HcRed1, respectively. The emission signal was detected using a VNP-29MC CCD detector (Vieworks, South Korea) with 3 × 3 pixel-shift modality enabled and with a triple-bandpass filter (470 ± 10, 540 ± 10 and 645 ± 50 nm) in the emission path.

To visualise the distribution of EPS in the biofilm we stained sialic acid and N-acetylglucosaminyl residues by supplementing solid M9 medium (0.2% glucose (w/v)) [35] with 20 μg/ml gentamicin and 2 μg/ml Alexa594-wheat germ agglutinin (WGA) (W11262, Invitrogen, USA) before inoculating with 1 × 10^4^ cfu/ml JM105-miniTn7-*gfp* and growing as previously described. We imaged EPS-stained specimens using widefield epi-fluorescence mesoscopy as before using a 490 nm LED to excite GFP and 580 nm LED to excite Alexa594-WGA.

We determined the lipid localisation throughout the biofilm by staining with Nile Red. We supplemented solid LB medium with 20 μg/ml gentamicin and 10 μg/ml Nile Red (72485, Sigma-Aldrich, USA) before inoculating with 1 × 10^4^ cfu/ml JM105-miniTn7-*gfp* and growing as previously described. We then imaged the lipid distribution in relation to the intra-colony channels using widefield epi-fluorescence mesoscopy as before using a 490 nm LED to excite GFP and 580 nm LED to excite Nile Red.

The protein distribution was determined by staining the biofilm with FilmTracer SYPRO Ruby biofilm matrix stain (F10318, Fisher Scientific, USA) which binds to a number of different classes of extracellular protein. Solid LB medium was prepared containing 20 μg/ml gentamicin and a final concentration of 2% (v/v) FilmTracer SYPRO Ruby biofilm matrix stain before inoculating with JM105-miniTn7-*gfp* and growing as previously described. Specimens were imaged using widefield epi-fluorescence mesoscopy. A 490 and 580 nm LED from a pE-4000 illuminator (CoolLED, UK) were used for GFP and SYPRO Ruby excitation, respectively. Fluorescence emission from GFP and SYPRO Ruby was detected as outlined above. Both channels were acquired sequentially.

### Disruption and recovery of intra-colony channel structures

To assess the ability of the structures we observe to recover following disruption, single colonies of JM105-miniTn7-*gfp* were grown on solid LB medium supplemented with 20 μg/ml gentamicin and allowed to grow for 10 h at 37 °C in darkened conditions. Following the initial growth step colonies were removed from the incubator and gently mixed with a sterile 10 μl pipette tip to disrupt the channel structures in the growing biofilm. Care was taken to prevent disruption to the underlying solid medium on which the colony was supported. Following disaggregation, the colonies were grown for a further 10 h at 37 °C in darkened conditions prior to imaging. Colonies were then mounted in sterile LB medium and imaged using widefield epi-fluorescence mesoscopy as described above. To determine the effect of channel disruption on the biofilm population, CFUs were numerated following disruption at regular intervals over a period of time. Briefly, JM105 macro-colonies were prepared as above. A proportion of the colonies were left undisturbed, while others were carefully mixed using a sterile 10 μl pipette tip every 40 min for a period of 8 h. To ensure adequate removal of the biofilm from the surface of the agar, a 6 mm cork-borer was used to punch our colonies and their surround medium before each plug was placed in an individual 2 ml aliquot of sterile LB broth and mixed vigorously for 15 s by vortexing. The cell suspensions were serially diluted and enumerated by spread plating on solid LB, before incubating at 37 °C for 16 h. Colonies were counted and the number of CFU/ml was calculated for each undisrupted and disrupted biofilm. An unpaired *t*-test was used to compare the change in CFU between each condition.

### Using differentially labelled isogenic strains to observe channels in mixed cultures

The phenomenon of strain sectoring has been previously documented and occurs by mechanical buckling as adjacent colonies expand into each other during radial growth [18, 19]. We investigated whether intra-colony channels were able to cross the strain boundary between sectors by inoculating a low-density mixed culture of JM105-miniTn7-*gfp* and JM105-miniTn7-*HcRed1* at a 1:1 ratio and inoculating a lawn onto solid LB medium containing 20 μg/ml gentamicin. We allowed colonies of each strain to stochastically collide into adjacent clonal populations during colony expansion and then imaged using widefield mesoscopy after incubation for 20 h at 37 °C in darkened conditions as described above. We used colony PCR to confirm that the miniTn7 insertion, which contained the photoprotein gene, occurred at the same chromosomal location in both strains (*glmS* Fwd.—5′ AAC CTG GCA AAT CGG TTA C; *tn7*R109 Rev.—5′ CAG CAT AAC TGG ACT GAT TTC AG). The miniTn7 transposon inserts at only one *attTn7* site in the chromosome, downstream of *glmS* [37]. We found that both JM105-miniTn7-*gfp* and JM105-miniTn7-*HcRed1* were inserted ~25 base pairs downstream of *glmS*. Therefore, there is no genotypic difference between the strains, save for the inserted photoprotein gene.

### Fluorescent microsphere uptake assay

To assess the function of the structures we observe, a confluent lawn of fluorescent microspheres was seeded along with the bacterial inoculum at the culturing stage. Two-hundred nanometre multi-excitatory microspheres (Polysciences, Inc., USA) were seeded at a density of 1 × 10^10^ microspheres/ml and plated along with 1 × 10^4^ cfu/ml JM105-miniTn7-*gfp* in a mixed-inoculum. Microsphere translocation was assessed by widefield epi-fluorescence mesoscopy as above with two-channel detection for both the GFP and microsphere fluorescence emission. A triple-bandpass emission filter that transmitted light at 470 ± 10, 540 ± 10 and 645 ± 50 nm was placed in the detection path. Sequential excitation of GFP and the fluorescent microspheres was achieved using a 490 and 580 nm LED, respectively, from a pE-4000 LED illuminator (CoolLED, UK) Each channel was acquired sequentially using a CCD camera detector (Stemmer Imaging, UK). All imaging was carried out using water immersion.

### Assessing the role of intra-colony channels in nutrient uptake

The functional role of the structures which we observe was tested using an arabinose biosensor where GFP expression was controlled by the presence or absence of L-arabinose. The biosensor strain contained the *araBAD* operon with *gfp-*inserted downstream on the promotor and ara*BAD* functional genes. The biosensor strain was a gift from colleagues at the James Hutton Institute.

JM105 transformed with the arabinose biosensor plasmid, pJM058, was grown overnight at 37 °C while shaking at 250 rpm in liquid LB medium supplemented with 25 μg/ml chloramphenicol. Overnight cultures were then diluted in fresh LB and grown until OD_600_ = 0.5. Cells were then pelleted and washed three times with 1x M9 salts. Washed cells were inoculated onto solid M9 minimal medium [35] with L-arabinose as the sole carbon source (0.2%) at a density of 1 × 10^4^ cfu/ml and grown for 42–48 h in darkened conditions at 37 °C. Specimens were then prepared for imaging as outlined above.

### Image processing and analysis

Widefield epi-fluorescence mesoscopy *z*-stacks were deconvolved where specified using with Huygens Professional version 19.04 (Scientific Volume Imaging, The Netherlands, http://svi.nl) using a Classic Maximum Likelihood Estimation algorithm. A theoretical point spread function was generated using Huygens Professional with parameters adjusted to suit the experimental setup. Deconvolution was performed using a server with a 64-bit Windows Server 2016 Standard operating system (v.1607), two Intel® Xeon® Silver 4114 CPU processors at 2.20 and 2.19 GHz and 1.0 TB installed RAM. Image analysis was performed using FIJI [38]. Figures presented here were linearly contrast adjusted for presentation purposes where required using FIJI [38].

## Results

### *E. coli* biofilms possess a network of intra-colony channels

**Fig. 1:**
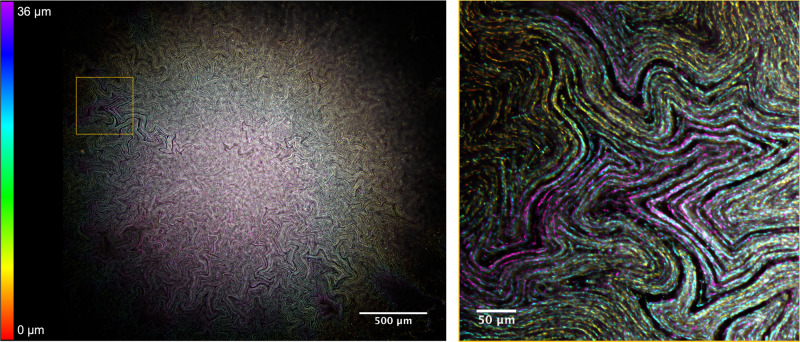
Visualising the intra-colony channel system of *E. coli* macro-colony biofilms. A deconvolved 36-μm-thick transverse sub-stack of a mature *E. coli* macro-colony biofilm acquired using widefield mesoscopy. An axial colour-coded LUT has been applied, which indicates the relative position of each cell within the context of the biofilm. A magnified ROI is presented where individual cells can be clearly resolved. Channel structures are seen to permeate throughout the biofilm and present a 3D topography within the context of the biofilm.

The internal architecture of *E. coli* macro-colony biofilms was investigated using conventional widefield epi-fluorescence microscopy, widefield mesoscopy and confocal laser-scanning mesoscopy. Using widefield mesoscopy it was apparent that *E. coli* (JM105) biofilms contain a network of channel-like structures which permeate the biofilm linking the centre of the colony to the leading edge. The channels measure ~15 μm wide and appear as non-fluorescing regions within the biofilm, which is lined by individual cells in a pole-to-pole arrangement. We applied a Classic Maximum Likelihood Estimation deconvolution algorithm to a sub-sample of a z-stack acquired using the Mesolens in widefield epi-fluorescence mode to improve image quality and reveal the arrangement of individual cells in a mature macro-colony biofilm. We then applied a colour-coded look-up table (LUT) according to the axial position of each optical section within the 36-μm-thick z-stack (Fig. 1). From the axial-coded LUT we can see that the intra-colony channels are not merely 2D lateral arrangements of cells, but that the channels have a 3D topography within the context of the biofilm, resembling canyons and ravines rather than enclosed capillaries.

Imaging of JM105 biofilms using confocal mesoscopy ensured that the deconvolution algorithm used to process widefield Mesolens data did not introduce erroneous structural artefacts. Confocal microscopy provides a marked improvement in signal-to-noise ratio compared with widefield techniques, particularly with thick specimens, resulting in a similar image quality to a deconvolved widefield dataset. Confocal mesoscopy revealed the same channel structures that we identified in widefield imaging experiments presented in Fig. 1 (Supplementary Fig. 2 and Supplementary Movie 1). This concludes that the structures we observed were not introduced as an artefact of image processing.

To demonstrate the benefit of using the Mesolens over conventional microscopes for imaging live biofilms, we also imaged biofilms using a conventional upright widefield epi-fluorescence microscope with a low magnification, low-NA lens (4×/0.13 NA). We compared the ability of the Mesolens and the conventional microscope to resolve the intra-colony channels and found that there was a clear improvement in the spatial resolution with the Mesolens (Supplementary Fig. 3). The resolution improvement applies to both lateral and axial resolution, and establishes the Mesolens as an ideal imaging technology for 3D imaging of large microbiological specimens with sub-micron resolution, enabling greater than single-cell resolution throughout the entire colony.

### Channels emerge as an inherent property of biofilm formation

**Fig. 2:**
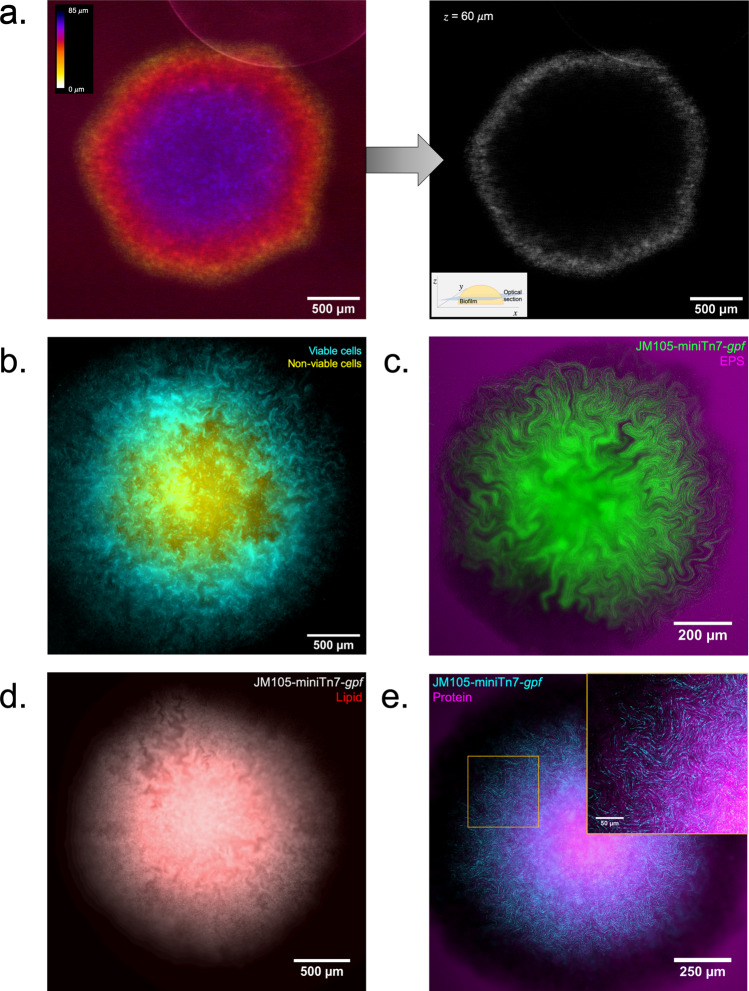
Characterising the structure of intra-colony channels. **a** Maximum intensity projection of an unlabelled JM105 colony acquired using reflection confocal mesoscopy, with a single isolated optical section shown. Reflection imaging determined that intra-colony channels were not occupied by material of differing refractive index to the biomass. The colony-medium interface can be observed clearly, while there is no evident structure within the colony. **b** Signal from non-viable cells (yellow) was subtracted from viable cells to negate any spectral overlap in the emission of Sytox Green and HcRed1. A composite maximum intensity projection of the entire colony is presented. Intra-colony channels in the viable cell population (cyan) did not contain any non-viable cells. **c** Alexa594-WGA-stained EPS residues (magenta) were not present in the intra-colony channels when compared with elsewhere in the biofilm, meaning channels were not composed of an EPS-based matrix. The high background signal in the surrounding agar is likely owed to non-specific binding of the WGA dye with gycan components of the agar substrate. **d** Nile red-stained lipids (red) clustered in the centre of *E. coli* biofilms while intra-colony channels remain unstained by Nile Red. Therefore, intra-colony channels were not composed of lipids. **e** Emission of SYPRO Ruby-stained extracellular proteins (magenta) mimicked the spatial patterns of intra-colony channels, showing that channels were filled by a protein-based matrix.

The channel structures we have identified appear as dark regions within the biofilm, and so we hypothesised that they may be comprised of a structural matrix. We began investigating the structural makeup of the channels by determining if they were filled with materials of differing refractive index compared with that of the biomass. Using reflection confocal mesoscopy, where signal is detected from reflections of incident light at refractive index boundaries, such as those between bacterial cells and the surrounding growth medium, we tested if the channels were comprised of translocated growth medium or air. A maximum intensity projection of an unlabelled *E. coli* JM105 biofilm acquired in reflection confocal mode showed no reflection signal resembling the intra-colony channels (Fig. 2a). This suggests that the channels must be of a similar refractive index to the surrounding biomass and biofilm matrix and are not occupied by solid growth medium or air.

To determine if the channel structures we observe were occupied by non-viable/non-fluorescing cells, biofilms were grown in the presence of the viability dye, Sytox Green. This dye has an emission peak at 523 nm enabling the use of HcRed1 *(λ*_*em*._ 618 nm) expressing JM105 *E. coli* cells for two-colour imaging. The false-coloured composite (Fig. 2b) shows a maximum intensity projection of a JM105-miniTn7-*HcRed1* biofilm stained with Sytox Green acquired using widefield mesoscopy, where live cells are presented in cyan and non-viable cells are shown in yellow. Subtracting the signal of the non-viable cells from HcRed1-expressing cells to prevent spectral overlap in the emission of the two fluorophores, such that no Sytox-labelled cells were falsely presented in cyan. It was observed that viable and non-viable cells formed two distinct domains within the colony. Here, non-viable cells cluster in the centre of the biofilm while intra-colony channels are not occupied by non-viable/non-fluorescent cells.

To determine if intra-colony channels were filled with exopolysaccharides (EPS) secreted by bacteria within the biofilm, JM105 biofilms were grown in the presence of the lectin-binding dye conjugate Alexa594-WGA. The deconvolved composite image of a JM105-miniTn7-*gfp* biofilm (green) and associated EPS (magenta) shows that EPS are distributed throughout the entire biofilm and are not strictly localised within the channel structures (Fig. 2c). Assessment of the lipid distribution using the lipid-binding dye Nile Red, showed that intra-colony channels are not composed of lipid (Fig. 2d). The protein-specific fluorescent dye, SYPRO Ruby revealed the presence of extracellular protein within the channels (Fig. 2e).

**Fig. 3:**
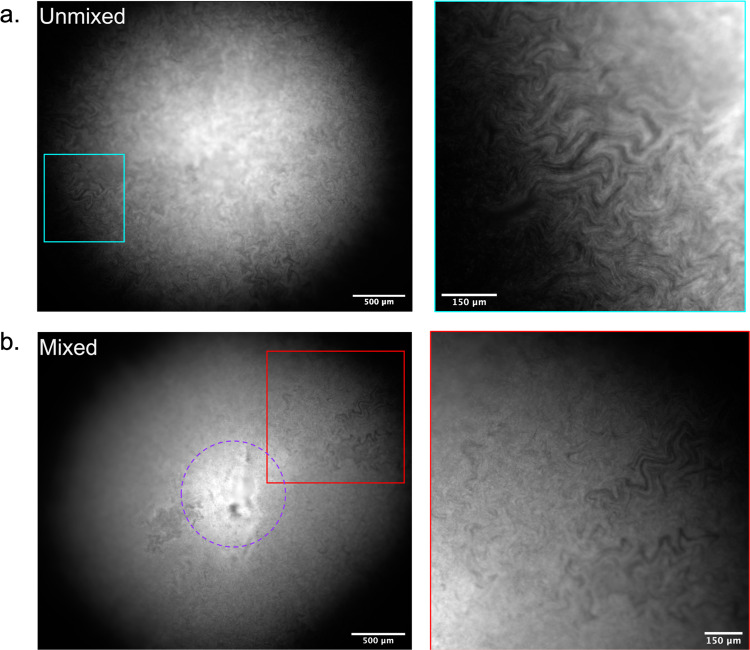
Intra-colony channels form as an emergent property of biofilm formation. **a** An unmixed, naïve control biofilm of JM105-miniTn7-*gfp* with established intra-colony channels. **b** A macro-colony JM105-miniTn7-*gfp* biofilm that was initially grown for 10 h before mechanical disruption and subsequent recovery and regrowth at 37 °C for a further 10 h. Regrowth was accompanied with the re-emergence of intra-colony channels in the outgrown region of the disrupted colony, showing that channel formation is an emergent property of macro-colony biofilm development. The purple circle indicates the boundary of the juvenile colony at the time of disaggregation, where channels have not reformed in the disrupted region.

To determine if the formation of intra-colony channels arose as an emergent property of biofilm formation, we tested if the structures were able to re-form following disruption. The colony biofilms were grown to the point of them establishing the formation of channels (Fig. 3a) and then the colony was mixed to create a uniform mass of cells. The colonies were then reimaged following a recovery period of 10 h. The channels reformed in the regions of the biofilm where new growth occurred (Fig. 3b). Interestingly, following continuous disruption over a number of hours, we found that there was a significant decrease (*P* value = 0.0423) in the number of CFU in the disrupted biofilms compared with the naïve biofilms (Supplementary Fig. 4). The ability of the channels to form in the same way as a naïve colony suggests that they form as an emergent property of *E. coli* colonial growth on a solid surface.

### Channels result in strain boundaries in mixed isogenic cultures

**Fig. 4:**
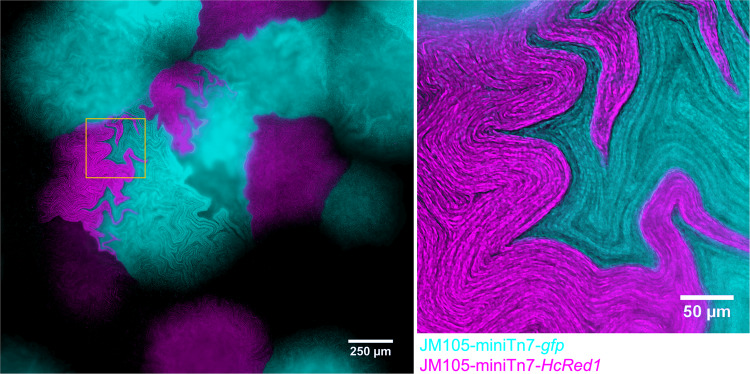
Intra-colony channels are confined within clonal populations and unable to cross strain boundaries. A mixed culture of isogenic JM105 strains which express either GFP (cyan) or HcRed1 (magenta). Each strain sectored into segregated clonal populations, which have propagated from a single colony-forming unit, and cells from each sector were unable to cross the strain boundary. The intra-colony channels present within each sector were also unable to cross the strain boundary and were therefore not shared by opposing isogenic colonies.

Growth of two isogenic strains in co-culture, each expressing a different photoprotein, results in sectoring and has been previously described [18–23]. We wished to explore this sectoring property in the context of intra-colony channel formation and to determine if the channels were shared between the strains. When the two isogenic strains sector, the channels do not intersect the boundary between the strains and are retained within a sector (Fig. 4). The confinement of channels was more evident between different populations (i.e., HcRed1 and GFP-expressing), whereas the boundaries between sectors of cells expressing the same photoprotein were less ordered.

### Intra-colony channels represent a novel nutrient acquisition system in *E. coli* biofilms

**Fig. 5:**
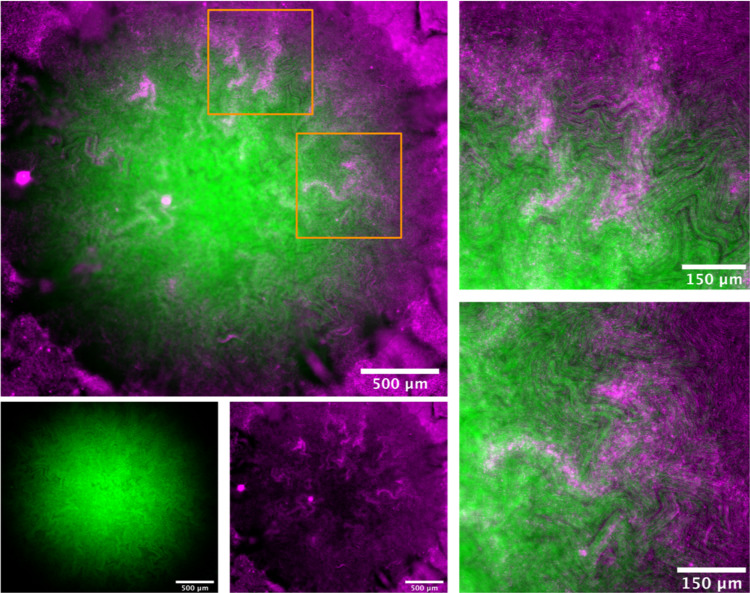
Intra-colony channels facilitate transport of microscopic particles. A single optical section ~25 μm above the base of the colony shows a mature JM105-miniTn7-*gfp* biofilm (green) and a lawn of 200 nm fluorescent microspheres (magenta). The fluorescent microspheres were transported from a confluent lawn at the base of the colony into the intra-colony channels and directed towards the centre of the colony. Two ROIs are presented from different regions of the colony where fluorescent microspheres were transported into the colony via intra-colony channels.

To investigate whether the intra-colony channels play a role in the transport of substances into the biofilm, the channel system was tested for functional roles by introducing 200 nm diameter fluorescent microspheres to the medium when preparing the specimen for widefield mesoscopy. The fluorescent microspheres were spread as a dense lawn along with a dilute mid-log JM105-miniTn7-*gfp* culture. A single optical section, 25 μm above the base of the colony, allows the outline of the colony to be observed at the edges of the image, with the untouched lawn of microspheres outside the colony (Fig. 5). The distribution of beads in these areas are homogenous, whereas within the colony the transport of the fluorescent microspheres through the channels reflects the spatial structure of the biofilm. Magnified regions of interest of intra-colony channels show that the channels are acting as conduits for the transport of microspheres into the biofilm. The transport of microspheres into the channels suggests that these intra-colony structures are involved in the acquisition of substances from the external environment. This suggests the ability of channels to transport small fluorescent particles could be extended to facilitate uptake of smaller particles and solutes into the colony, and may represent a previously unknown nutrient acquisition system in microbial assemblages.

**Fig. 6:**
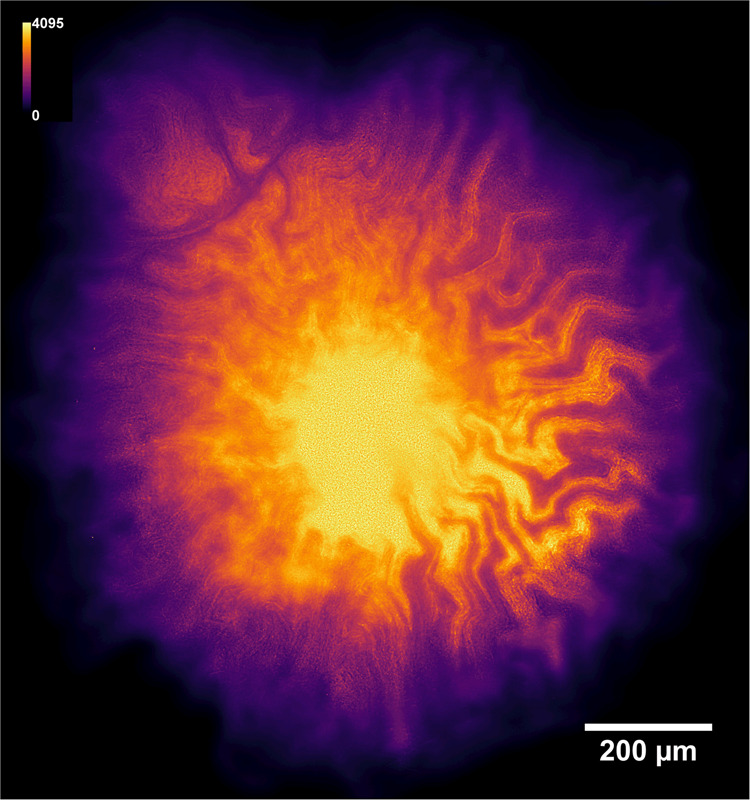
Intra-colony channels play a functional role in nutrient acquisition and transport to the centre of bacterial biofilms. A deconvolved image of a JM105-pJM058 macro-colony biofilm grown on M9 minimal medium with L-arabinose as the sole carbon source. This arabinose biosensor expresses GFP only in the presence of L-arabinose. GFP emission intensity was higher in cells, which line the intra-colony channels compared with cells elsewhere within the biofilm, which shows that the channel structures have a higher concentration of L-arabinose compared with elsewhere within the biofilm. This provides evidence of a functional role in nutrient acquisition and transport for the intra-colony channel system.

To further investigate the role of intra-colony channels in biofilm nutrient acquisition, an arabinose-inducible GFP strain (*E. coli* JM105 P_BAD_*-gfp*) was utilised. Growth of the arabinose-inducible GFP strain on solid minimal medium with L-arabinose as the sole carbon source revealed that the biofilm fluoresced most intensely in regions surrounding the intra-colony channels (Fig. 6). This suggests that the concentration of L-arabinose is highest within the channels compared with the remainder of the biofilm and demonstrates the role of these structures act as a nutrient acquisition and transport mechanism within the colony. This finding challenges the long-held belief that bacterial colony nutrient uptake occurs through passive diffusion from the basal or apical surface of the biofilm through the extracellular matrix, and concurs with previous hypotheses which suggested that large biofilms must develop transport mechanisms to direct nutrients to their centre [1].

## Discussion

This study is the first application of the Mesolens to microbiology and has offered a new approach for imaging large microbial specimens, enabling us to characterise a novel and hitherto unseen structural aspect of *E. coli* macro-colony biofilms. This emergent functional property of biofilm growth enables nutrient acquisition and transport in these large microbial assemblages and was observed in every macro-colony examined by mesoscopy. Previous biofilm imaging studies have mainly used conventional widefield and laser-scanning microscopy to study biofilm architecture, which are inherently limited by sacrificing spatial resolution and imaging volume. For example, automated tile-scanning microscopes which change the location of the FOV or focal plane have been used to image growing colonies from 1 × 10^1^ to 1 × 10^4^ cells [39–41]; however, this method often requires long acquisition periods and results in tiling artefacts. With the Mesolens we negate the need for stitching and tiling when imaging multi-millimetre specimens and can image beyond small bacterial aggregates to visualise live bacterial macro-colonies in excess of 1 × 10^9^ cells while maintaining sub-micron resolution throughout the entire 6 mm^2^ field. Therefore, in comparison with other conventional large specimen imaging techniques, the Mesolens stands as a novel and improved method for in situ imaging of live bacterial communities. In addition, recent advances in light sheet microscopy [42] and mutli-photon microscopy [43, 44] have been applied to biofilm imaging. However, these methods currently cannot resolve sub-micron information over multi-millimetre scales, as with the Mesolens. The same problem accompanies ultrasound [45, 46], optical coherence tomography and photoacoustic tomography [47–49] methods used for mesoscale biofilm imaging, where they cannot properly resolve structures on the order of which we report. We have also studied images of bacterial macro-colonies under a widely available conventional stereomicroscope. Careful comparison with Mesolens images suggests that traces of the channel may be faintly visible in spite of the low resolution of stereomicroscopes in *x*, *y* and particularly *z* dimensions.

The structures we have identified bear similarities to some other aspects of bacterial community architecture, however it is important to note that the channels we identify are fundamentally different to structures. For example, the water irrigation channels discovered in mushroom-shaped *Pseudomonas* and *Klebsiella spp*. biofilms, which differ from intra-colony channels we observe by their location at the base of large submerged microbial aggregates and often limited to biofilms under flow conditions [31, 50]. There have also been channel-like structures identified in mature bacterial colonies, such as crenulation in *B. subtilis* macro-colonies [29, 30] or the macroscopic folds of *P. aeruginosa* biofilms [27, 28]. It is important to note, that crenulations and folds are all visible as surface structures of the colony and resolvable using photography techniques, whereas the intra-colony channels identified here are present within the main body of the biofilm and are not observable by viewing the surface of the colony. A similar phenomenon was recently reported in colonies of *Proteus mirabilis* where 100-nm-diameter fluorescent microspheres were observed to penetrate the boundary of the colony through ‘crack-like conduits’ present at the colony edge [51]. However, the authors were unable to resolve any spatial evidence of the conduits themselves.

The spatial arrangement of the intra-colony channels is fractal in nature, with repeating patterns and complex topographies. Upon first glance, channels resemble fractal features found in multi-strain colonies, which form as a result of the mechanical instability between growth and viscous drag of dividing cells [19]. However, these features have only been reported in multi-strain colonies where the fractal dendrites have been composed of live, fluorescing cells [20–23, 52]. The spatial patterns we observe are different to those shown previously. First, the patterns we observe arise within a single population of cells where there are no strain-to-strain interactions to result in the formation of fractal patterns. Given that the intra-colony channels are not occupied by dead non-fluorescing cells (Fig. 2b) it is clear that the bacterial colonies used in this work are not composed of two pseudo-domains (i.e., viable and non-viable cells) which could interact to form complex 3D fractal patterning. Our finding that non-viable cells localise in the centre of the biofilm agrees with previous studies showing that dense microbial aggregates often have dense hypoxic, acidic centres which have diminished access to nutrients [11, 12, 43, 53–57].

The intra-colony channels form as an inherent property of biofilm formation, leading to fractal-like patterns that exhibit plasticity, which is reminiscent of the results of a classical eukaryotic developmental biology experiment by Moscona, where reformation of the channel architecture in marine sponges occurred after disaggregation by passage through a fine silk mesh [58, 59]. The ability of the channels to re-form also suggests that they fulfil a functional role in the context of biofilm biology.

In summary, we have identified a previously undocumented nutrient uptake system in colonial biofilms which challenges the current belief that cells which are out with the reach of underlying nutrient-rich medium are able to gain nutrients beyond diffusion through a homogenous mass of cells their exopolymeric matrix [14–17]. While we have observed these channel structures in several *E. coli* K-12 isolates, it is currently unknown if these channels are limited to only *E. coli* or if they are a widely conserved feature of large microbial aggregates. To assess this requires further study involving a number of phylogenetically diverse strains and subsequent investigation into factors such as cell shape, cell–surface interactions and division mechanics to determine the processes that guide channel formation. Furthermore, quantitative analysis of these structures is made difficult by the large file size of Mesolens data (i.e., a full-volume three-channel image stack ≈1.5 TB). Consequently, these data are generally incompatible with most freely available image analysis programmes. Nevertheless, the presence of these channels may represent a route to acquire nutrients throughout complex environmental assemblages of organisms. Moreover, these channels may represent a route to circumvent the chemical protection and resistance phenotype of bacterial biofilms [60], such that rather than relying on antibiotics to penetrate biofilms by diffusion, it may be possible to exploit the intra-colony channels for delivery of antimicrobial agents. Although these observations were made under standard laboratory conditions, given the conserved structure of microbial communities in the environment, the occurrence of these channel structures may also be widespread under environmental conditions. The formation of these channels in the environment would also serve as a potential mechanism to explain how nutrient transport occurs in large microbial communities in nutrient limited conditions. This has yet to be explored in an environmental setting, however the findings presented here offer an overview of how to visualise and resolve these structures by means of optical mesoscopy. This could be further developed to help understand the ecological impact of our observations as the drivers of biofilm formation a natural context are poorly understood [61]. Aside from identifying and characterising intra-colony channels, this study has established the Mesolens as a much needed and powerful tool for studying microbial communities, and by extension could be applied to any aspect of microbial ecology, environmental biology and pathogen microbiology. Ultimately, the identification and characterisation of an intra-colony channel network could therefore have far-reaching applications while providing further understanding on the acquisition of nutrients by microbial communities.

## Supplementary information

Supplementary information

Supplementary movie 1
